# Neurotoxicity Prediction of Compounds: Integrating Knowledge-Guided Graph Representations with Machine Learning Approaches

**DOI:** 10.3390/ijms27083543

**Published:** 2026-04-16

**Authors:** Yongxin Jiang, Yilin Gao, Yi He, Shu Xing, Weiwei Han

**Affiliations:** Key Laboratory for Molecular Enzymology and Engineering of Ministry of Education, School of Life Sciences, Jilin University, Qianjin Road 2699, Changchun 130012, China; jiangyx23@mails.jlu.edu.cn (Y.J.); gaoyl2321@mails.jlu.edu.cn (Y.G.); heyi21@mails.jlu.edu.cn (Y.H.)

**Keywords:** neurotoxicity, large language model, machine learning, SHAP interpretability, drug development

## Abstract

Neurotoxicity from drugs and environmental pollutants poses serious risks to brain function, yet existing computational models mainly target general neurotoxicity and lack specialized tools for brain-specific assessment. This study aimed to develop and validate a high-performance, brain-focused neurotoxicity prediction framework to improve drug safety evaluation and toxicity screening. We systematically analyzed molecular features, clustering patterns, and target predictions of brain-toxic compounds. Multiple feature representations were compared, including traditional molecular fingerprints, knowledge-guided pre-trained graph Transformer (KPGT) embeddings, and transformer-based MolFormer embeddings, combined with machine learning classifiers. Model performance was evaluated using multiple metrics, and SHAP analysis was conducted to identify influential molecular substructures. Toxic molecules showed physicochemical properties favoring central nervous system (CNS) penetration, including lower molecular weight, lower LogP, fewer hydrogen bond donors/acceptors, fewer rotatable bonds, and lower polar surface area (PSA). The KPGT-MLP model achieved the best balanced performance, with an accuracy (ACC) of 0.8928 and an ROC-AUC of 0.9459, clearly outperforming traditional fingerprint-based models, MolFormer-based models, and general prediction tools such as DI-NeuroT and ADMETlab 3.0. Overall, this study establishes a robust framework for brain-specific neurotoxicity prediction, with the KPGT-MLP model demonstrating strong accuracy and robustness. The proposed approach provides an effective strategy for early neurotoxicity screening and risk assessment, offering valuable insights for safer drug design and advancing computational toxicology and drug discovery.

## 1. Introduction

Drug-induced Neurotoxicity, or the detrimental effects of drugs on the brain, can range from mild cognitive impairments to severe neurodegenerative diseases. These toxic reactions not only severely impact patients’ quality of life but may also limit the clinical use of otherwise effective medications. For example, chemotherapeutic agents such as methotrexate and cisplatin are known to cause cognitive declines, referred to as “chemobrain” [1,2]. However, the neurotoxicity of chemotherapy drugs extends beyond cognitive decline, presenting a broad spectrum of clinical syndromes including acute, subacute, and chronic encephalopathies, posterior reversible encephalopathy syndrome, acute cerebellar dysfunction, meningitis, and neurovascular syndromes. These clinical entities vary in etiology, severity, progression, and timing of occurrence [3]. Additionally, some commonly used medications have also been shown to be associated with long-term cognitive decline, such as Benzodiazepines and anticholinergic drugs [4,5,6], which may be linked to dementia. Cefepime has been implicated in causing altered mental states and non-convulsive seizures in patients [7]. These observations underscore the importance of assessing neurotoxicity in drug development.

Early identification of drugs that may cause neurotoxicity can significantly reduce treatment failures due to severe neurological side effects and decrease the economic losses associated with drug withdrawal or restricted use. Despite the existence of various neurotoxicity assessment methods, such as animal and cell models, there are limitations to their inability to accurately mimic human responses. Significant variability exists between humans and experimental animals in brain gene expression profiles, neurotransmitter receptor profiles [8], and the regulatory pathways involved in neuron regulation and migration [9]. Compared to the complexities of the central nervous system, cell models are overly simplistic, and traditional 2D cultures may not fully predict toxicological responses due to the lack of a 3D environment and in vivo processes [10,11]. Emerging 3D human brain organoids also face issues such as the lack of models with higher degrees of maturation, fewer cell types, and potentially overlooking communications between the brain and other organs [12]. Development is still in its early stages and they have not yet been widely applied. Therefore, there is an urgent need to develop reliable and efficient computational approaches that can complement experimental methods and enable large-scale, accurate prediction of neurotoxicity.

In recent years, advances in computational methods have significantly accelerated the development of predictive models in drug discovery and toxicology. Traditional cheminformatics approaches, such as molecular fingerprints and descriptor-based representations, have been widely adopted due to their simplicity and interpretability. However, these handcrafted features rely on predefined rules and often fail to capture the complex structural and contextual information inherent in molecular representations. Consequently, their ability to generalize across diverse chemical spaces and accurately model intricate structure–activity relationships remains limited.

In parallel with the development of traditional cheminformatics tools, the rapid progress of large language models (LLMs) has introduced new opportunities in fields such as natural language processing, urban computing, and scientific discovery. Recent studies, such as POI-Enhancer for geospatial representation and LLM4SD for scientific rule extraction, have demonstrated LLMs’ powerful capabilities in feature representation and knowledge inference. Inspired by this trend, several recent efforts have attempted to apply LLMs to chemical domains, leveraging molecular SMILES representations as input sequences for extracting latent semantic features of molecules to support classification and property prediction tasks.

However, general-purpose LLMs are often extremely large in parameter scale and require extensive computational resources, making them impractical for tasks that demand efficiency and scalability, such as large-scale toxicity screening or molecular regression modeling. In response to this challenge, our study incorporates lightweight, chemically pre-trained language models, including ChemBERTa, MolFormer, and SMILES-BERT, as molecular feature extractors. ChemBERTa, based on a BERT-like architecture, is pre-trained on large-scale SMILES corpora and offers effective molecular semantic encoding with significantly reduced model size and inference cost. Unlike general LLMs, these models can be efficiently integrated into conventional machine learning workflows and run on local hardware.

In recent years, computational methods have increasingly been used in toxicological assessments of drugs, offering an efficient and cost-effective alternative. However, specialized tools for predicting neurotoxicity specifically targeted at neurotoxicity are still scarce. Existing computational models such as ADMETlab generally focus on toxicity, not specifically on neurotoxicity [13]. Given that the blood–brain barrier (BBB) acts as a natural protective membrane that prevents toxins and pathogens in the bloodstream from entering the central nervous system (CNS) and also limits the penetration of most chemical compounds into the brain [14], molecules involved in neurotoxicity differ from general toxic compounds and do not completely overlap. Furthermore, the identification of brain-toxic molecules in environmental pollutants, such as pesticides and organophosphates, is increasingly important due to the significant public health risks they pose. Organophosphates, widely used in pesticides, have been shown to cause neurotoxic effects, leading to both acute and chronic neurological disorders. This highlights the urgent need for accurate predictive models that can assess the Neurotoxicity risks of both pharmaceutical compounds and environmental contaminants.

Existing computational frameworks for neurotoxicity prediction, such as DINeuroTpredictor [15], have been developed primarily based on traditional machine learning approaches using molecular descriptors or fingerprint-based features. While these models have demonstrated reasonable predictive performance, their reliance on handcrafted features limits their ability to capture complex molecular patterns and subtle structure–activity relationships associated with neurotoxicity. In recent years, advances in deep learning have provided more powerful representation learning strategies, enabling models such as graph neural networks and transformer-based architectures to learn richer and more expressive molecular features directly from data. In particular, the emergence of large language models, which treat molecular SMILES as sequences, has opened new avenues for capturing semantic and contextual information in molecular structures. Building on these developments, knowledge-guided pre-trained frameworks such as KPGT further enhance molecular representation by integrating domain knowledge with large-scale pretraining, offering improved performance and generalization for neurotoxicity prediction tasks.

**Figure 1:**
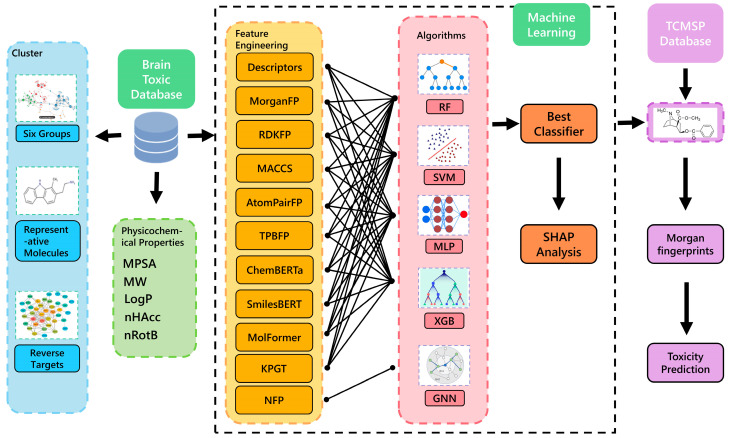
Workflow of the study. The diagram illustrates the overall process, including data collection, feature extraction, model construction, and application.

In this study, we employ the latest KPGT (Knowledge-Graph-based Pretrained Transformer) technology combined with advanced machine learning algorithms, such as Multi-Layer Perceptron (MLP), to develop a predictive model for neurotoxicity. By leveraging KPGT’s ability to generate deep molecular embeddings from SMILES strings, we enhance the representation of molecular structures, which are then used as input features for the MLP model. Positive neurotoxicity data were obtained from the Comparative Toxicogenomics Database (CTD) for model training and learning [16]. This approach allows us to capture complex molecular interactions and improve the accuracy of neurotoxicity predictions. Our model, trained and validated on a large-scale chemical database, demonstrates higher predictive accuracy than traditional models, achieving an accuracy of 89.28% and an AUC of 0.9459. Additionally, we have identified major chemical groups associated with neurotoxicity, providing vital guidance for the safety design of future drugs. The workflow of our study is illustrated in Figure 1.

## 2. Results and Discussion

### 2.1. Molecular Feature Evaluation of the Dataset

**Figure 2:**
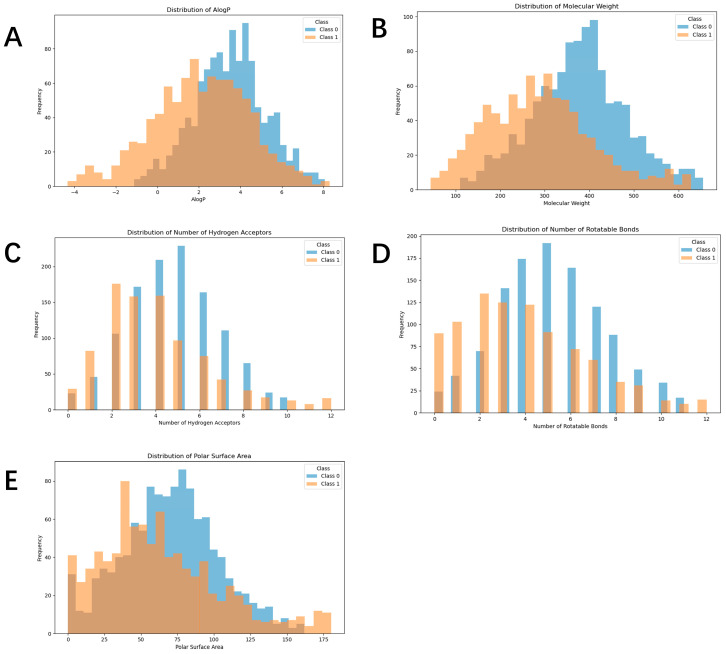
Physicochemical properties of negative and positive datasets. (**A**) AlogP, (**B**) molecular weight, (**C**) number of hydrogen bond acceptors, (**D**) number of rotatable bonds, and (**E**) polar surface area (PSA). Orange indicates positive samples, and blue indicates negative samples.

Studies have found that drugs adhering to Lipinski’s Rule of Five (Molecular weight ≤ 500, Oil/water distribution coefficient (LogP) ≤ 5, Hydrogen bond donors ≤ 5 (expressed as the sum of OHs and NHs), and Hydrogen bond acceptors ≤ 10 (expressed as the sum of Ns and Os), with a later addition of a fifth rule: the number of rotatable bonds ≤ 10) are more likely to be permeable and absorbed [17,18]. Furthermore, drugs that satisfy the following criteria of a molecular weight ≤ 400, LogP ≤ 5, Hydrogen bond donors ≤ 3, Hydrogen bond acceptors ≤ 7 [17], and a Polar Surface Area (PSA) less than 60–70 are more likely to exhibit central nervous system penetration [19], meaning they can potentially cross the blood–brain barrier. Physicochemical property analysis of the collected brain toxic drugs reveals that toxic drugs, in comparison to non-toxic ones, have a lower molecular weight, lower LogP, fewer hydrogen bond donors, fewer rotatable bonds, and a lower PSA. These are all indicators of better CNS activity in the molecules (Figure 2A–E).

### 2.2. Clustering

**Figure 3:**
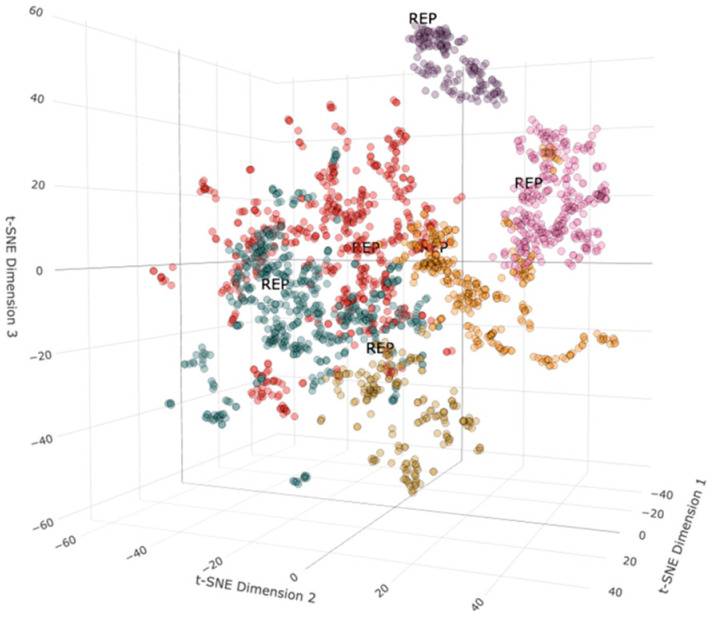
Cluster Analysis of 231 brain toxic molecules. Points of the same color represent the same cluster.

By examining the feature distribution of toxic and non-toxic molecules, we found that non-toxic molecules have a narrower distribution in chemical space compared to toxic molecules. This suggests that toxic molecules may share some common characteristics. However, due to the substantial overlap of positive and negative data in the chemical space, dimensionality reduction alone cannot effectively distinguish between toxic and non-toxic molecules. This necessitates the exploration of machine learning and deep learning techniques. The presence of multiple small clusters indicates that these molecules may induce Neurotoxicity through different mechanisms. Therefore, we performed hierarchical clustering analysis on the brain toxic molecules (Figure 3). The 231 molecules analyzed in Figure 3 are all brain-toxic, and the clustering analysis focuses on their characteristics. Representative molecules for each cluster were identified through similarity analysis. As shown in the figure, The molecules were grouped into six clusters. The structures of all small molecules are preserved in the Supplementary Materials.

### 2.3. Target Prediction

**Figure 4:**
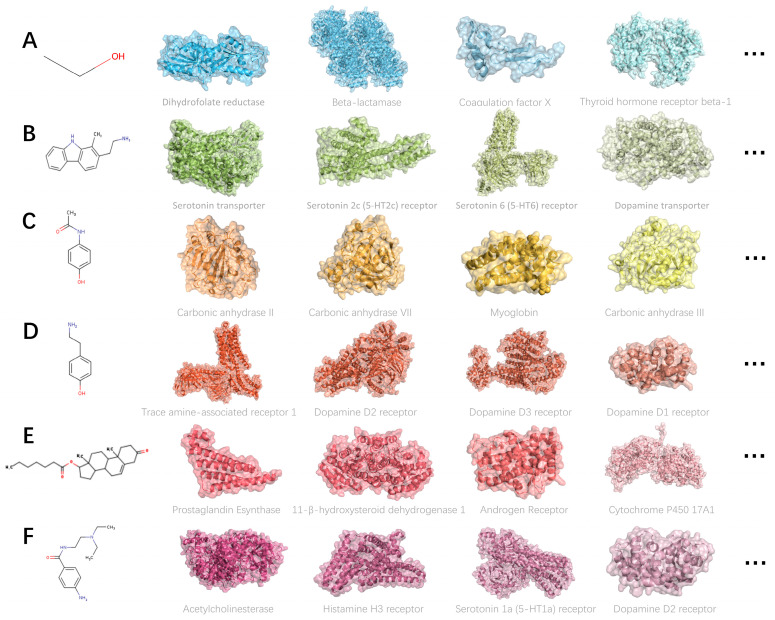
Reverse target prediction of representative molecules from six classes. (**A**) CCO (SMILES), (**B**) 2-ethanamine, (**C**) acetaminophen, (**D**) tyramine, (**E**) NSC12872, and (**F**) procainamide. Proteins shown in the same color indicate that they are the targets of the corresponding molecule on the left.

We used SwissTarget Prediction to predict the targets of six representative molecules and analyzed the molecular mechanisms underlying their toxicity. The targets of each molecule are shown in Figure 4. We analyzed the top 50 targets for each molecule, and the results are also depicted in Figure 4. The targets for molecule CCO include enzymes, phosphatases, and p450, which may affect the intracellular environment. Molecule 2-ethanamine targets lyases and kinases, potentially impacting signaling pathways. The targets for molecule Acetaminophen include G protein-coupled receptors, ion channels, membrane proteins, and nuclear receptors. These six molecules exhibit distinct mechanisms of action with minimal overlap in targets, highlighting the complexity of Neurotoxicity mechanisms.

### 2.4. Machine Learning and Deep Learning

**Figure 5:**
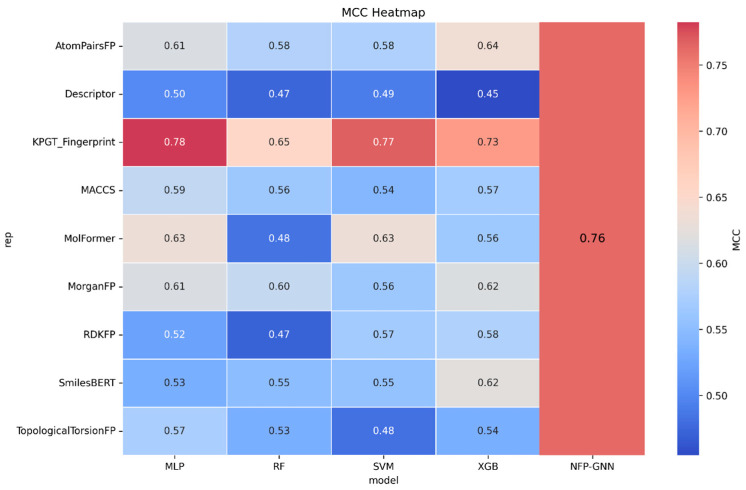
The accuracy of machine learning and deep learning.

A comprehensive analysis of the data presented in Table 1 and Figure 5 reveals substantial variation in predictive performance across different combinations of molecular fingerprints/descriptors and downstream classifiers. Notably, KPGT paired with either Support Vector Machine (SVM) or MLP demonstrates markedly superior performance, substantially outperforming all other model combinations across nearly all key evaluation metrics.

Specifically, the KPGT-based models achieve specificity (SP) in the range of 0.81–0.90, Matthews correlation coefficient (MCC) approaching 0.78, F1 score stably around 0.81–0.87, balanced accuracy (BA) of 0.82–0.89, and ROC-AUC values between 0.91 and 0.95. This consistently strong and well-balanced performance indicates that the knowledge-guided pre-trained graph Transformer representations extracted by KPGT provide significantly more discriminative molecular features for the present binary classification task than traditional molecular fingerprints and most self-supervised language model approaches, establishing KPGT as the most effective and robust feature extraction strategy among those evaluated.

Ranking closely behind are the MolFormer series representations, particularly when coupled with SVM or MLP, which deliver highly competitive results approaching those of KPGT. These combinations attain SE of 0.79–0.80, MCC ≈ 0.63, F1 score ≈ 0.79–0.80, and ROC-AUC in the 0.89–0.90 range—clearly surpassing the best results obtained with most conventional fingerprints. However, MolFormer representations exhibit pronounced sensitivity to the choice of downstream classifier: performance deteriorates sharply when using RF or XGBoost, with MCC values dropping to the 0.48–0.56 range. This suggests that while MolFormer generates high-quality embeddings, their effective utilization relies heavily on linear or shallow nonlinear classifiers, in contrast to the greater classifier robustness observed with KPGT.

Traditional molecular fingerprints generally underperform relative to the advanced learned representations. Among them, AtomPairsFP remains the strongest performer, achieving SE ≈ 0.805, MCC ≈ 0.615–0.637, and F1 ≈ 0.79 when paired with XGBoost or MLP, offering reasonable overall balance. The best results from other well-known fingerprints (MorganFP, MACCS, RDKFP, etc.) typically fall within a narrower and lower range of MCC 0.56–0.62 and F1 0.77–0.79. TopologicalTorsionFP and especially pure physicochemical descriptors exhibit clearly inferior performance, with descriptor-based models consistently ranking at the bottom across all classifier combinations. This indicates that conventional descriptor-based feature engineering provides limited information gain for the neurotoxicity prediction task under study.

The SmilesBERT family shows moderate overall performance. Even when paired with its best-performing classifier (XGBoost), it reaches an MCC of only ≈0.62 and a maximum F1 score of ≈0.80, lagging behind both MolFormer and KPGT in discriminative power and balance, although it still outperforms most traditional fingerprints. These results suggest that autoregressive language models trained primarily on SMILES strings still possess considerable room for improvement in molecular property prediction tasks—at least under the current pre-training scale and fine-tuning strategies—compared with graph-structure-aware models (such as KPGT) or more recent chemistry-oriented language models (such as MolFormer).

In summary, when the primary objective is to maximize predictive performance and robustness, the KPGT + MLP combination is recommended as the preferred modeling approach. The KPGT-MLP model consistently excels across all major performance metrics—including sensitivity, specificity, MCC, accuracy, precision, F1 score, balanced accuracy, and AUC—demonstrating a clear and substantial advantage in the prediction of neurotoxicity, positioning it as one of the most promising predictive models currently available in this domain.

### 2.5. Comparison with Other Models

**Figure 6:**
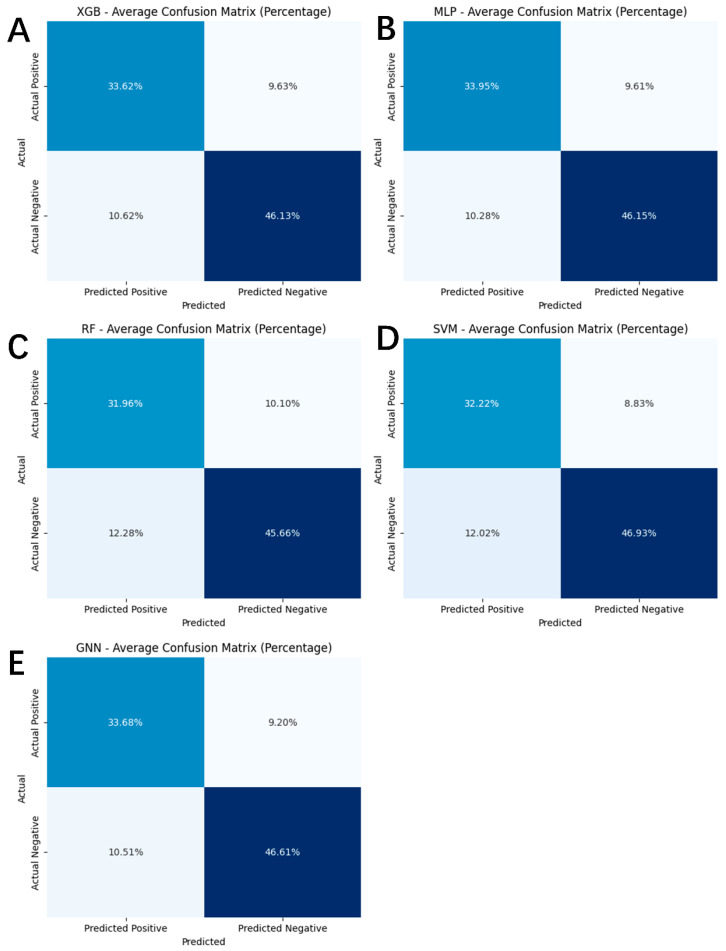
Confusion matrix of various models: XGB (**A**), MLP (**B**), RF (**C**), SVM (**D**), GNN (**E**).

Figure 6 presents the confusion matrices of the various models and Table 2 compares the performance of the KPGT-MLP model with two representative neurotoxicity prediction models across multiple evaluation metrics, including Sensitivity (SE), Specificity (SP), Matthews Correlation Coefficient (MCC), Accuracy (ACC), Precision (P), F1 Score (F1), and Balanced Accuracy (BA). It is important to note that Neurotoxicity represents a specific subtype of neurotoxicity, primarily affecting the central nervous system (CNS), particularly the brain. Due to the current lack of publicly available models specifically designed for Neurotoxicity prediction, we selected DINeuroT and ADMETlab3.0—both general neurotoxicity models—as comparative benchmarks. Although these models are not explicitly focused on brain-specific effects, they offer the most relevant references available for comparative assessment.

Specifically, the KPGT-MLP model performed exceptionally well across several metrics, achieving SE of 0.8830, SP of 0.9004, MCC of 0.7825, ACC of 0.8928, P of 0.8737, F1 of 0.8783, and BA of 0.8917. In contrast, DINeuroT shows substantially weaker performance, with low SE (0.5882), SP (0.4706), and an MCC of only 0.0592, indicating poor discriminative ability. ADMETlab3.0 attains high sensitivity (0.9167) but suffers from very low specificity (0.3083), suggesting a strong bias toward false positive predictions, which is further reflected in its moderate MCC (0.2753) and BA (0.6125). Overall, the KPGT-MLP model exhibits the most robust and reliable predictive performance among all compared methods, highlighting its clear advantage for neurotoxicity prediction tasks.

Overall, the KPGT-MLP model shows considerable potential in predicting the neurotoxicity of newly synthesized or discovered molecules. It is trained on molecular representations derived from SMILES strings, making it applicable to compounds with known chemical structures. The KPGT framework generates generalized molecular embeddings through large-scale pretraining and knowledge-guided representation learning, which enables the model to capture underlying structural patterns and enhances its generalization capability for previously unseen compounds. However, the model’s predictive performance for novel molecules may depend on their similarity to the chemical space represented in the training dataset. Compounds with substantially different structural characteristics or those lying outside the scope of the training data may lead to decreased prediction accuracy. Therefore, while the model exhibits strong generalization potential, further validation using external datasets and prospective experimental studies is crucial to fully assess its applicability in real-world scenarios.

### 2.6. Analysis of Key Features Identified by SHAP

**Figure 7:**
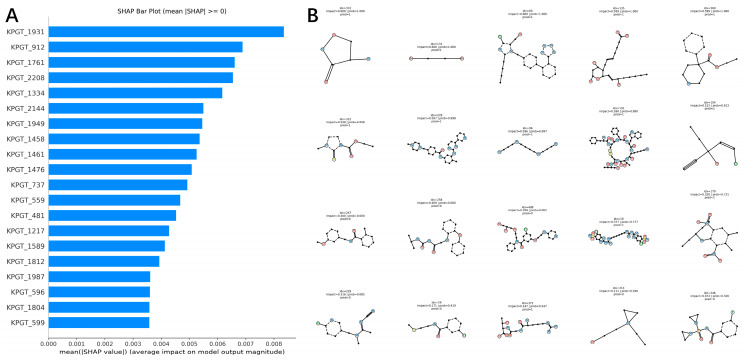
SHAP analysis of the KPGT-MLP model. (**A**) SHAP bar plot showing the mean absolute SHAP values of KPGT embedding features, indicating their relative importance in the model. (**B**) Representative molecular structures of test samples with the highest overall SHAP impact, illustrating examples where the model relies most strongly on learned embedding features.

Figure 7 provides a SHAP-based interpretability analysis of the KPGT-MLP model from both feature-level and sample-level perspectives. Panel A shows the mean absolute SHAP values of KPGT embedding dimensions, revealing that a subset of latent features, such as KPGT_1931, KPGT_912, and KPGT_1761, contributes most significantly to the model predictions. The relatively smooth decrease in SHAP values across the top-ranked features suggests that the model relies on multiple embedding dimensions with distributed contributions, rather than a single dominant feature.

Panel B presents representative test molecules with the highest cumulative SHAP impact. These molecules exhibit considerable structural diversity, including both simple and complex scaffolds, indicating that strong model responses are not limited to a specific class of compounds. Instead, the model appears to leverage patterns encoded in the learned representation space across chemically diverse structures. This observation further suggests that the model is capable of generalizing across different molecular forms rather than relying on narrowly defined structural motifs.

Taken together, these results suggest that the KPGT-MLP model captures informative patterns within the latent embedding space and utilizes a combination of influential features to drive predictions. However, since KPGT features are abstract representations, the analysis reflects model behavior in the embedding space rather than direct associations with specific chemical substructures. Additionally, the use of representative high-impact samples provides a complementary perspective on model behavior, highlighting cases where the learned representations contribute most strongly to prediction outcomes.

## 3. Materials and Methods

### 3.1. Data Collection

The positive dataset for toxic encephalopathy was collected from the Comparative Toxicogenomics Database (CTD, https://ctdbase.org/, accessed on 28 January 2026) [16], and the negative dataset was gathered from Pubchem (https://pubchem.ncbi.nlm.nih.gov/, accessed on 28 January 2026) [17]. The positive and negative datasets consist of 948 and 1196 molecules, respectively, represented in SMILES format. The molecular structures within the dataset were processed using the standardizer package (version 0.1.9), which involves the removal of counterions, solvent fractions, and salts, followed by the addition of hydrogen atoms to obtain a standardized set of molecule SMILES data.

### 3.2. Molecular Feature Evaluation of the Dataset

We utilized the RDKit cheminformatics library (version 2020.03.1) to parse the SMILES strings of each molecule in the dataset and convert them into molecular objects [19]. Using the Descriptors module and the MoleculeDescriptors module in RDKit, a series of molecular descriptors were calculated, including molecular weight (MolWt), topological polar surface area (TPSA), number of hydrogen bond donors (NumHDonors), number of hydrogen bond acceptors (NumHAcceptors), LogP (MolLogP), and molecular volume (MolMR). After calculating the properties for all molecules, we normalized all molecular descriptors using the StandardScaler. Distribution plots for each property were then created using the histplot function from the Seaborn library (version 0.13.2), with molecules categorized as toxic or non-toxic based on their classification. The diversity of chemical structures is a critical factor in building machine learning models. Principal Component Analysis (PCA) is a commonly used method for dimensionality reduction [20].

### 3.3. Clustering Analysis of Small Molecule Data

To analyze the clustering of small molecule similarities, we used molecular fingerprints, dimensionality reduction, clustering, and 3D visualization. Morgan fingerprints were computed for each compound using RDKit, with a radius of 2 and 2048 bits [21]. SMILES strings were converted to molecular objects for fingerprint generation, excluding invalid ones. Duplicates were removed to ensure uniqueness in the combined dataset. We applied t-SNE with a perplexity of 30 to reduce the fingerprint data to three dimensions [22]. Hierarchical clustering using Ward’s method was performed on the t-SNE results, setting the number of clusters to 6. Each compound was assigned a cluster label. Within each cluster, we calculated a similarity matrix using the Tanimoto coefficient and identified the molecule with the highest average similarity as the representative [23]. A 3D scatter plot was then created with plotly [24], coloring each compound by cluster assignment and distinctly marking representative molecules, enabling intuitive exploration of small molecule similarities and relationships. Next, we conducted a comprehensive evaluation and prediction for the six representative small molecules, using SwissTarget (https://swisstargetprediction.ch/, accessed on 30 January 2026) to predict potential targets [25].

### 3.4. Machine Learning Models

In this study, we loaded data from CSV files and preprocessed it to remove any missing values. The dataset comprised SMILES strings representing molecular structures and their corresponding toxicity classification labels. To characterize the molecules, we used five different types of molecular fingerprints and a set of molecular descriptors as features, all calculated using the open-source software RDKit (version 2020.03.1). Specifically, the molecular fingerprints used included the following: Morgan fingerprints (1024 bits) [21], which are similar to Extended Connectivity Fingerprints (ECFP) and capture circular substructures [26]; RDK fingerprints, which are path-based and capture substructure information based on molecular paths; MACCS keys (166 bits) [27], a predefined set of structural keys commonly used in cheminformatics for rapid screening; AtomPairs fingerprints (1024 bits) [28], which encode pairs of atoms and their topological distances to describe relationships within the molecular structure; and Topological Pharmacophore Atom Triplets (TPBFP, 1024 bits) [29], which encode triplet interactions based on pharmacophore features within the molecule. Additionally, we selected a set of 208 RDKit molecular descriptors (referred to as RDKitDes) to develop descriptor-based predictive models [30]. In addition to traditional molecular fingerprints and descriptors, we incorporated transformer-based molecular embeddings derived from the large language model to enhance molecular representation. Specifically, we used the pre-trained ChemBERTa-zinc-base-v1 model (version 1.0), MolFormer-XL-both-10pct model and SMILES_BERT model from the Hugging Face repository (https://huggingface.co/, accessed latest on 2 April 2026), implemented using transformers (version 4.38.2) and PyTorch (version 2.2.0) SMILES strings were tokenized with the associated tokenizer, and the [CLS] token embedding from the last hidden state was extracted as the molecular vector. In this study, we also utilized the KPGT framework to enhance molecular feature representation for toxicity classification. These embeddings were then used as input features for machine learning models such as Random Forest and MLP to perform toxicity classification. To ensure reproducibility and robust evaluation, the dataset was split into training and test sets using a stratified sampling strategy to preserve the class distribution. Specifically, 80% of the data was used for model training and 20% was held out as an independent test set. Within the training set, hyperparameter tuning was performed using k-fold cross-validation (k = 5), where the training data was further divided into five folds. In each iteration, four folds were used for training and one fold for validation. This process was repeated across all folds, and the average performance was used to select optimal model configurations. Hyperparameter optimization for all models was conducted using a grid search strategy. A predefined set of candidate hyperparameters was systematically evaluated, and the best combination was selected based on the highest mean cross-validation performance. The area under the receiver operating characteristic curve (ROC-AUC) was used as the primary scoring metric during model selection, as it provides a threshold-independent measure of classification performance, particularly suitable for imbalanced datasets. These descriptors included properties such as molecular weight, LogP, polarizability, number of rings, and counts of hydrogen bond donors and acceptors.

To improve predictive performance, we selected four different machine learning models and optimized the hyperparameters for each model. First, the Random Forest (RF) model [31], an ensemble recursive partitioning method, was optimized using five hyperparameters: the number of trees (n_estimators: 200, 500), maximum tree depth (max_depth: None, 10, 20), minimum number of samples per leaf (min_samples_leaf: 1, 2, 4), and the number of features considered at each split (max_features: “log2”, “auto”, “sqrt”). The Random Forest model enhances prediction accuracy and robustness by constructing multiple decision trees, each built from a random subset of the training data.

Second, the SVM model determines the optimal hyperplane in the feature space by maximizing the margin between classes [32], thus distinguishing objects with different labels. We optimized two hyperparameters for the SVM: the kernel function coefficient (gamma: ‘scale’, ‘auto’) and the penalty parameter for error terms (C: 0.01, 0.1, 1, 10). The SVM model is particularly suited for high-dimensional spaces and can handle nonlinear classification problems by mapping data to higher-dimensional spaces to find the optimal separating hyperplane.

Third, the MLP is a type of artificial neural network used for complex pattern recognition [33]. We adjusted hyperparameters including hidden layer sizes (hidden_layer_sizes: (128,), (256,), (128, 64)), activation functions (activation: ‘tanh’, ‘relu’), solvers (solver: ‘sgd’, ‘adam’) and initial learning rates (learning_rate_init: 0.001, 0.01). The MLP captures complex patterns and relationships through multiple hidden layers and nonlinear activation functions.

Finally, the Extreme Gradient Boosting (XGBoost) model [34], an advanced ensemble learning method based on the gradient boosting framework, was optimized with hyperparameters including maximum tree depth (max_depth: 3, 5, 7). The XGBoost model improves performance by incrementally adding new trees to correct errors made by previous trees.

During model training and evaluation, we employed several key performance metrics, including the Area Under the ROC Curve (ROC-AUC) [35], Sensitivity (SE), Specificity (SP), Matthews Correlation Coefficient (MCC), Accuracy (ACC), Precision (P), F1 Score (F1), and Balanced Accuracy (BA). The AUC provides a comprehensive measure of model classification performance across all possible thresholds. Sensitivity (SE) measures the model’s ability to correctly identify true positive cases, while Specificity (SP) measures its ability to correctly identify true negative cases. MCC takes into account all four categories of the confusion matrix, assessing the quality of binary classifications and ranging from −1 (complete misclassification) to 1 (perfect classification). Accuracy is the proportion of correctly classified samples, while Precision is the proportion of true positives among predicted positives. The F1 Score is the harmonic mean of precision and recall, making it useful for imbalanced datasets. Balanced Accuracy is the average of SE and SP, providing a fair evaluation for imbalanced classes. These metrics allow us to comprehensively assess each model’s performance in predicting Neurotoxicity, ensuring the accurate identification of both positive and negative samples.

### 3.5. GNN Model

The Graph Neural Network (GNN) was developed employing the PyTorch framework [36]. This implementation leverages a WeaveGNN architecture [37], as provided by the Deep Graph Library (DGL) [38], to facilitate enhanced feature extraction regarding potential interactions between atoms and bonds. The network architecture incorporates hidden layers with a feature dimensionality of 128, utilizes the ReLU activation function, and comprises two graph convolution layers. A sophisticated readout mechanism employing both weighted summation and maximization strategies is followed by a linear layer containing 128 neurons and a final output layer. Furthermore, the model employs a learning rate of 0.001, the Adam optimization algorithm, a cross-entropy loss function, and an early stopping mechanism to mitigate the risk of overfitting.

## 4. Conclusions

This study systematically investigated the molecular characteristics, clustering behavior, target predictions, and model performance of brain-toxic compounds, establishing a robust framework for neurotoxicity prediction. The results indicate that compounds conforming to Lipinski’s Rule of Five tend to exhibit physicochemical properties favorable for central nervous system penetration, including lower molecular weight, reduced LogP, fewer hydrogen bond donors, fewer rotatable bonds, and lower polar surface area. Among the evaluated feature representations, KPGT-derived embeddings significantly outperformed traditional molecular fingerprints, with the KPGT-MLP model achieving the most balanced and superior performance across key metrics (ACC 0.8928; ROC-AUC 0.9459), clearly surpassing existing tools such as DINeuroT and ADMETlab 3.0. SHAP analysis further revealed critical molecular substructures influencing predictions, providing valuable insights for toxicity-aware drug design. Although MolFormer-based representations showed slightly lower performance than KPGT, they demonstrated competitive results while reducing reliance on hand-crafted features, highlighting their potential for scalable and high-throughput applications.

Despite the promising performance of the proposed framework, several limitations should be acknowledged. First, the model was developed and validated on a specific dataset, which may limit its generalizability to broader chemical spaces, particularly for novel compounds with structures that are underrepresented in the training data. Second, the positive endpoint used in this study, namely toxic encephalopathy, captures only a subset of central nervous system (CNS) toxicity. Neurotoxicity is a multifaceted phenomenon encompassing diverse phenotypes, and future work should extend the modeling framework to include additional neurotoxicity endpoints for a more comprehensive assessment. Third, although the KPGT-based representation demonstrated superior predictive capability, its reliance on pre-trained embeddings may introduce biases inherited from the underlying training corpus. Fourth, the current study primarily focuses on classification performance and does not yet incorporate mechanistic biological information or in vivo validation, both of which are essential for fully understanding neurotoxicity. Additionally, the proposed prediction framework has not been validated by researchers external to this project. External validation will be an important direction for our future work. Finally, although the model shows strong predictive performance, its applicability in real-world drug development pipelines requires further validation on fully independent external datasets and prospective studies.

In summary, this study presents a powerful framework for neurotoxicity prediction by integrating advanced deep learning-based molecular representations with machine learning classifiers. The KPGT-MLP model stands out for its exceptional accuracy and reliability, offering a promising approach for future drug discovery and modern toxicological evaluation.

## Figures and Tables

**Table 1:** Comparison of models metrics.

Model	SP	MCC	ACC	P	F1	BA	AUC
SmilesBERT-XGB	0.8494	0.6209	0.8135	0.8022	0.7849	0.8089	0.8577
SmilesBERT-RF	0.8075	0.5506	0.7786	0.7540	0.7480	0.7748	0.8381
SmilesBERT-MLP	0.8201	0.5349	0.7716	0.7584	0.7337	0.7653	0.8623
SmilesBERT-SVM	0.8159	0.5548	0.7809	0.7609	0.7487	0.7764	0.8455
MolFormer-XGB	0.8117	0.5650	0.7855	0.7606	0.7566	0.7822	0.8681
MolFormer-RF	0.7866	0.4834	0.7459	0.7213	0.7078	0.7407	0.8380
MolFormer-SVM	0.8326	0.6320	0.8182	0.7917	0.7958	0.8163	0.9000
MolFormer-MLP	0.8368	0.6316	0.8182	0.7947	0.7947	0.8158	0.8909
KPGT-XGB	0.8714	0.7262	0.8648	0.8385	0.8474	0.8639	0.9306
KPGT-RF	0.8133	0.6548	0.8275	0.7794	0.8112	0.8295	0.9118
KPGT-SVM	0.8963	0.7682	0.8858	0.8677	0.8700	0.8843	0.9538
KPGT-MLP	0.9004	0.7825	0.8928	0.8737	0.8783	0.8917	0.9459
MorganFP-XGB	0.8410	0.6165	0.8112	0.7946	0.7840	0.8073	0.8943
MorganFP-RF	0.8577	0.5967	0.8019	0.8035	0.7658	0.7947	0.8937
MorganFP-SVM	0.8494	0.5633	0.7855	0.7882	0.7444	0.7773	0.8663
MorganFP-MLP	0.8117	0.6148	0.8089	0.7727	0.7887	0.8085	0.8865
MACCS-XGB	0.8243	0.5688	0.7879	0.7705	0.7560	0.7832	0.8713
MACCS-RF	0.8410	0.5633	0.7855	0.7816	0.7473	0.7784	0.8644
MACCS-SVM	0.8326	0.5442	0.7762	0.7701	0.7363	0.7689	0.8569
MACCS-MLP	0.8285	0.5930	0.7995	0.7796	0.7713	0.7958	0.8689
Descriptor-XGB	0.7406	0.4547	0.7296	0.6869	0.7010	0.7282	0.8189
Descriptor-RF	0.7741	0.4749	0.7413	0.7112	0.7056	0.7370	0.8337
Descriptor-SVM	0.8075	0.4917	0.7506	0.7371	0.7068	0.7432	0.8311
Descriptor-MLP	0.8159	0.5011	0.7552	0.7457	0.7107	0.7474	0.8493
RDKFP-XGB	0.8577	0.5777	0.7925	0.7988	0.7521	0.7841	0.8677
RDKFP-RF	0.8285	0.4719	0.7413	0.7453	0.6838	0.7300	0.8365
RDKFP-SVM	0.8619	0.5684	0.7879	0.8000	0.7437	0.7783	0.8544
RDKFP-MLP	0.8033	0.5214	0.7646	0.7432	0.7292	0.7596	0.8389
AtomPairsFP-XGB	0.8326	0.6369	0.8205	0.7927	0.7990	0.8189	0.8968
AtomPairsFP-RF	0.8159	0.5794	0.7925	0.7672	0.7652	0.7895	0.8720
AtomPairsFP-SVM	0.8452	0.5776	0.7925	0.7886	0.7562	0.7858	0.8752
AtomPairsFP-MLP	0.8117	0.6148	0.8089	0.7727	0.7887	0.8085	0.8762
TopologicalTorsionFP-XGB	0.8159	0.5352	0.7716	0.7556	0.7351	0.7658	0.8624
TopologicalTorsionFP-RF	0.8452	0.5345	0.7716	0.7771	0.7247	0.7621	0.8485
TopologicalTorsionFP-SVM	0.8326	0.4816	0.7459	0.7516	0.6895	0.7347	0.8427
TopologicalTorsionFP-MLP	0.8201	0.5741	0.7902	0.7688	0.7606	0.7864	0.8541

ACC: Accuracy; AUC: Area Under the Curve; F1: F1 Score; MCC: Matthews Correlation Coefficient; MLP: Multi-Layer Perceptron; P: Precision; RF: Random Forest; SP: Specificity; SVM: Support Vector Machine; XGB: eXtreme Gradient Boosting.

**Table 2:** Comparison of our model metrics and other models.

Model	SE	SP	MCC	ACC	P	F1	BA
KPGT-MLP	0.8830	0.9004	0.7825	0.8928	0.8737	0.8783	0.8917
DINeuroT	0.5882	0.4706	0.0592	0.5294	0.5263	0.5556	0.5294
ADMETlab3.0	0.9167	0.3083	0.2753	0.5787	0.5146	0.6592	0.6125

## Data Availability

The original contributions presented in this study are included in the article/Supplementary Material. Further inquiries can be directed to the corresponding authors.

## Supplementary Materials

The following supporting information can be downloaded at: https://www.mdpi.com/article/10.3390/ijms27083543/s1.

## Abbreviations

The following abbreviations are used in this manuscript:

KPGT	Knowledge-guided Pre-trained Graph Transformer
LLMs	Large Language Models
BBB	Blood–brain Barrier
CNS	Central nervous system
MLP	Multi-Layer Perceptron
CTD	Comparative Toxicogenomics Database
MolWt	Molecular Weight
TPSA	Topological Polar Surface Area
NumHDonors	Number of Hydrogen Bond Donors
NumHAcceptors	Number of Hydrogen Bond Acceptors
MolMR	Molecular Volume
PCA	Principal Component Analysis
SVM	Support Vector Machine
XGBoost	Extreme Gradient Boosting
AUC	Area Under the ROC
SE	Sensitivity
SP	Specificity
MCC	Matthews Correlation Coefficient
ACC	Accuracy
P	Precision
BA	Balanced Accuracy
GNN	Graph Neural Network
DGL	Deep Graph Library
PSA	Polar Surface Area
