# Supplementary figures and images for: Neurotoxicity Prediction of Compounds: Integrating Knowledge-Guided Graph Representations with Machine Learning Approaches
Source: Int J Mol Sci. 2026 Apr 16;27(8):3543. doi: 10.3390/ijms27083543 (PMC13115767; doi:10.3390/ijms27083543)

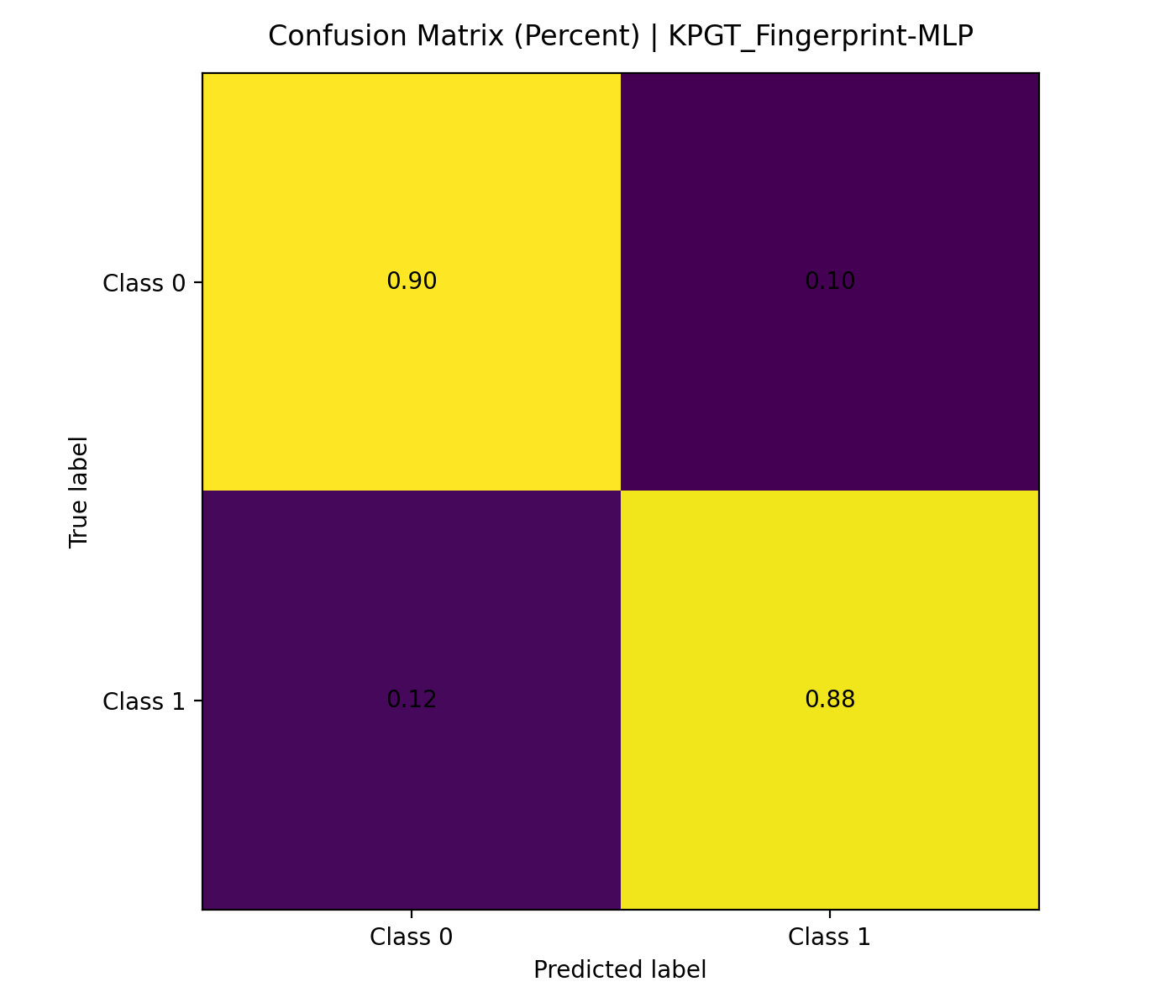

Supplement: Supplementary file 1 [file ijms-27-03543-s001.zip › KPGT_Fingerprint-MLP/confusion_matrix_percent.png]
